# Algae and the city: the genetic and ecophysiological diversity of photobionts in two areas of Madrid (Spain) with contrasting levels of nitrogen pollution

**DOI:** 10.1007/s11356-025-36681-0

**Published:** 2025-07-09

**Authors:** Francisco Gasulla, Leonardo M. Casano, Sergio Pérez-Ortega

**Affiliations:** 1 Department of Life Sciences, Universidad de Alcala, Alcala de Henares, Spain; 2 Department of Mycology, Real Jardín Botánico (CSIC), Madrid, Spain

**Keywords:** Chlorophyll fluorescence, Desiccation-tolerance, Lichen, Osmotic stress, Photobiont diversity, *Trebouxia*

## Abstract

Diverse hypotheses have been proposed to explain the low diversity and abundance of lichens in large cities, including high nitrogen pollution and the heat-island effect. Furthermore, it remains unclear which partner, the mycobiont or the photobiont, is more affected by these stresses that limit lichen survival. This study aimed to explore how nitrogen pollution and xeric conditions affect lichen photobionts. Photobionts from 25 lichen species in seminatural and urban areas of Madrid (Spain) were identified by amplifying the nrITS barcode region. Sixteen species-level strains were detected, including 6 new species-level lineages. Four photobiont strains were selected for physiological experiments based on their hosts' environmental preferences: *Trebouxia* A74, found only in urban lichens; *T. jamesii*, found only in semi-natural environments; and *T*. I01 and *T. gigantea*, found in both environments. These strains were cultured in vitro, and their photosynthetic activity was monitored under varying concentrations of KNO₃ and (NH₄)₂SO₄ for 12 weeks, with biomass quantified at the end of the experiment. Additionally, the photosynthesis response to long-term desiccation was analysed over 15 months. *T. jamesii* was negatively affected by exposure to nitrogen compounds and desiccation. In contrast, low concentrations of ammonium and nitrate stimulated growth in the other species, especially in *T*. A74, while desiccation even improved the photosynthetic performance of *T*. I01 and *T. gigantea*. These findings demonstrate that different *Trebouxia* phycobionts exhibit distinct species-specific physiological traits that may influence their lichen hosts' ability to survive in urban environments.

**Supplementary Information:**

The online version contains supplementary material available at 10.1007/s11356-025-36681-0.

## Introduction

In recent decades, European city centers have shown a reversal in the extension of"lichen deserts"—areas where no lichen species were found—with these zones becoming smaller or even disappearing (Hawksworth 2002; Munzi et al. 2007). The steady decline in SO₂ emissions has enabled lichen species, absent for decades or even centuries, to recolonize urban areas (European Environmental Agency 2010). However, the urban lichen biota is significantly reduced compared to that of natural environments, dominated by a high proportion of nitrophytic species, i.e., species with a certain degree of tolerance to nitrogen pollution (Van Dobben and Ter Braak 1998; Ruoss 1999; Loppi et al. 2002; Wolseley and James 2002). At present, the burning of fossil fuels in vehicles and heating systems is the main source of pollution in urban areas (Davies et al. 2007). Due to the high temperatures reached in the engines, the combustion of fuels can produce nitrogen oxides (NO_x_) and ammonia (NH_3_) (Frahm et al. 2009; Bobbink et al. 2010), which can be deposited as dry particles. In the presence of water, these compounds are converted to nitrate ions (NO_2_^−^) and ammonium (NH_4_^+^), respectively. Lichens lack roots, active gas exchange structures and protective tissues such as the cuticle of plants. As a result, the thallus absorbs water, nutrients and gases directly from the atmosphere and precipitates them over its entire surface. Several studies have reported a significant positive correlation between total nitrogen concentration in lichens and exposure to traffic-related N pollution (Coffey and Fahrig 2012; Gombert et al. 2003) or to agricultural activities (Ruoss 1999; Botersdorf and Werner 2013). Nitrogen is essential for the metabolism of lichen bionts. Therefore, exposure to low concentrations of nitrogen compounds can be beneficial and can increase photosynthetic capacity (Palmqvist et al. 1998; Gaio-Oliveira et al. 2005b; Palmqvist and Dahlman 2006). However, as the contamination load increases, nitrogen compounds accumulate in the lichens, which can become harmful. Depending on the tolerance limits, lichens are commonly classified as oligotrophic, with low tolerance to nutrients such as nitrogen compounds, and eutrophic, with high tolerance to nutrients, especially nitrogen (Hauck 2010). Accordingly, the proximity to the nitrogen source increases the abundance of nitrophytic species, while oligotrophic species show the opposite trend (i.e., De Bakker 1989; Van Herk 1999, 2001; Sparrius 2007).

An alternative or complementary hypothesis to explain lichen biota composition in cities is that it is more influenced by the harsh conditions of urban environments than by pollution. Large cities have heat islands at their centres, which are characterised by high temperatures and low air humidity. This, in turn, reduces the frequency of condensation phenomena such as dew (Kim 1992). Most nitrophytic lichens are also classified as xerophytic species (Nimis 2025). In 1969, Rydzak proposed the drought hypothesis, which was promptly criticized by several authors due to the accumulation of data demonstrating the negative effects of SO_2_ in lichens (i.e. Gilbert 1970; Coppins 1973; LeBlanc and Rao 1973; Fields 1988). This hypothesis was forgotten for decades until new experimental evidence and field data reopened the discussion, concluding that lichens are affected by both air pollutants and the relatively drier conditions of urban areas (Tretiach et al. 2007a,b; Käffer et al. 2011; Piccotto et al. 2011; Coffey and Fahring 2012; Tretiach et al. 2012; Delgado-Baquerizo et al. 2014; Munzi et al. 2014a). Urban lichens undergo short hydration periods, as a result, they have less time to repair cellular damage caused by dryness and/or pollution (Riddell et al. 2008; Carter et al. 2017). Piccotto et al. (2011) and Tretiach et al. (2012) found that xerophytic lichens are more abundant in cities due to their powerful antioxidant mechanisms and high recovery capacities, which help them survive prolonged dryness. These mechanisms can also counteract the toxic effects of pollution. In addition, Frahm (2013) proposed that the accumulation of dry N deposition may act like salt, causing an osmotic stress to the lichen symbionts. It has been observed that eutrophic lichens have a higher intracellular osmotic pressure than oligotrophic lichens, which enables eutrophic lichens to withstand the negative effects of salinity from N deposition and water shortage (Frahm 2013).

Another controversial question is which of the symbiont—the fungus or the photosynthetic partner- is more sensitive to urban abiotic stresses and therefore determines the sensitivity of the entire symbiosis. Numerous studies have been conducted on this topic; however, depending on the species surveyed, the type of treatment, or the analytical techniques employed, it is possible to conclude that either the mycobiont or the photobiont is more sensitive. On one hand, the higher sensitivity of the mycobiont to contaminants could be due to its greater surface exposure (Gaio-Oliveira et al. 2005a and references therein). Similarly, Munzi et al. (2009) concluded that, since the mycobiont represents the vast majority of a lichen biomass, the electrolyte leakage from lichen thalli caused by high N doses must be mostly due to damage in the fungal cells. Other authors have found that the the mycobiont limits the tolerance to N, as lichen species with the same photobiont have different sensitivities to N (Pirintsos et al. 2009; Munzi et al. 2014a, b; Munzi et al. 2017). In this regard, Maslaňáková et al. (2015) reported that two lichens, *Cladonia arbuscula* subsp. *mitis* and *Cladonia furcata,* responded differently to excess nitrogen despite having the same photobiont, although the identity of the photobiont was assumed and not molecularly tested. Proteomic analysis performed on *Cladonia portentosa* exposed to an excess of nitrogen also showed that the mycobiont is more affected by N than the photobiont, as most relevant changes in protein expression were observed in the fungal partner, whereas the photobiont mainly supports the defense mechanisms initiated by the mycobiont with an increased energy production (Munzi et al. 2017). On the contrary, other authors have suggested that the photobiont partner is more sensitive to N pollution than the mycobiont. For instance, Hauck (2010), in a comprehensive review regarding the ammonium and nitrate tolerance in lichens, concluded that “*the tolerance to high nitrogen levels depends, among others, on the capability of the photobiont to provide sufficient amounts of carbon skeletons for ammonia assimilation*”. Several studies have shown a negative correlation between photosynthetic activity and the levels of N pollution (von Arb etal 1990; Munzi et al. 2010; Munzi et al. 2014a, b). Langmann et al. (2014) observed that net photosynthetic rates were progressively reduced in lichens exposed to diesel exhaust while dark respiration was barely affected, indicating a greater effect on the photobiont than on the mycobiont. A decrease in photosynthetic activity may compromise the response of the entire thallus to nitrogen pollution by reducing the capacity to synthesize carbon skeletons.

The diversity of photobionts associated with urban lichens has rarely been addressed. Although photobiont type is commonly used as a functional trait in ecological studies of pollution in urban environments using lichens (e.g. Koch et al. 2019; Vieira et al. 2018), photobiont identity has rarely been studied at the species or strain level. Several studies have observed a reduced diversity of photobionts associated with urban or polluted environments (Ohmura et al. 2006; Rola et al. 2021). However, in some cases, this correlation was not found (Bačkor et al. 2010; Osyczka et al. 2021). Therefore, there is a lack of knowledge regarding the diversity of photobionts in lichen symbioses in urban environments. Additionally, there is a need for physiological studies to examine the effects of N pollution on photobionts at the species and/or strain level.

The current study aims to address the impact of nitrogen pollution on urban lichen biota, with a specific focus on the photobiont. We hypothesize that photobiont diversity and physiology are shaped by the contrasting environmental conditions in urban and semi-natural areas, particularly by nitrogen compounds such as NH_4_^+^ and NO_3_. Specifically, we propose that (1) photobionts associated with lichens in urban environments exhibit a reduced diversity compared to those in semi-natural areas, and (2) nitrogen pollution has a detrimental effect on photosynthetic performance and growth, which may be exacerbated under osmotic stress or arid conditions characteristic of urban areas. To test these hypotheses, photobionts were cultured and identified at the molecular level from lichen samples collected from semi natural and urban environments in the metropolitan area of Madrid (Spain). Subsequently, the physiological responses of isolated photobionts to increasing concentrations of NH_4_^+^ and NO_3_, and osmotic stress were examined, as well as their capacity to recover photosynthetic activity following prolonged desiccation after nitrogen treatments.

## Material and methods

### Lichen sampling

A total of 25 lichen species were collected from two areas within the Madrid municipality, each with contrasting nitrogen deposition levels. The semi-natural area (SN) is located in Monte de El Pardo, which covers approximately 15,289 hectares of Mediterranean forest. The forest is primarily composed of holm oak (*Quercus rotundifolia*) and maritime pine (*Pinus pinaster*), with a few other tree species such as cork oak (*Quercus suber*), Portuguese oak (*Quercus faginea*), and Western prickly juniper (*Juniperus oxycedrus*) in smaller numbers. This area is protected as a Special Protection Area. The mean air pollution dataset from a proximate station, despite being situated in an urban area, demonstrated an average hourly concentration of 18 μg/m^3^ and a maximum of 100.6 μg/m^3^ of NO_2_ for the 2015–2017 period (https://airedemadrid.madrid.es/). Samples were collected at 40.511531º N, 3.750402º W, 679 m above the sea level (a.s.l.). A total of 17 lichen species were collected at this location (see Suppl. Table 1). The urban area (U) is located in the centre of Madrid, and corresponds to two green areas/public gardens at a distance of c. 200 m from each other (40.406164° N, −3.685784° W, 630 m.a.s.l., and 40.411208° N, −3.682781° W, 689 m. a.s.l.). The mean air pollution dataset from two proximate stations, showed an average hourly concentration of 46.16 μg/m^3^ and a maximum of 272.16 μg/m^3^ of NO_2_ for the 2015–2017 period (https://airedemadrid.madrid.es/). Ten lichen species were collected from these two locations (see Suppl. Table 1). Among all collected lichens, only two species occurred in both the semi-natural forest and the city centre. The collected material was air-dried in the shade for one day and stored at −20 °C until needed for experimentation. Vouchers of all specimens were deposited at MA-Lichen, the lichen herbarium of the Real Jardín Botánico (Madrid, Spain). Species nomenclature of lichen-forming fungi follows Italic 8.0 (Nimis 2025).

### Isolation and identification of the photobionts

We assessed the diversity of lichen photobionts using a combined approach. First, we attempted to isolate photobionts from one lichen thallus of each species collected in SN and U. Second, we studied the diversity of photobionts associated with each species by amplifying the nrITS region in several thalli of each taxon. To isolate the photobiont, we followed the method described by Gasulla et al. (2010) to obtain axenic cultures of lichen photobionts. After 45 days of growth, ten colonies were selected under a stereo-microscope and subcultured onto Petri dishes containing 1.5% agar 3NBBM medium supplemented with glucose (20 g L^−1^) and casein (10 g L^−1^) (*Trebouxia* medium, Ahmadjian 1967) using a sterile toothpick. To identify each colony molecularly, the same toothpick was immersed in a microcentrifuge tube containing the PCR reaction mix. The mix consisted of 0.5 µl (10 mM) of each of the primer pair ITS1T -ITS4T (Kroken & Taylor 2000), 6.5 µl of MyTaq Mix (Bioline), which contains MyTaq DNA Polymerase and dNTPs, and 7.5 µl of distilled water. PCR amplifications were carried out under the following conditions: an initial 4 min denaturation phase at 95ºC, followed by 30 cycles of 1 min at 94ºC, 1 min 15 s at 52ºC and 1 min 30 s at 72ºC, and a final extension step of 7 min at 72ºC.

To assess the diversity of photobionts in lichen thalli in both areas, we extracted the complete lichen DNA using either the Speedtools Tissue DNA Extraction kit (Biotools) or a modified version of the CTAB method (Cubero et al. 1999) and amplified the barcode region nrITS as above, using two μl of DNA. The PCR products were purified using ExoSAP-IT™ PCR. Two µl of DNA were amplified employing the same primers, PCR reaction mix, and conditions as described above. The PCR products were purified using ExoSAP-IT™ PCR Product Cleanup Reagent (ThermoFisher) following the manufacturer's instructions. Macrogen Inc. (Madrid, Spain) sequenced the products using the same primer set as for PCR amplification. The sequence contigs were assembled using SeqMan v.12.0 (Lasergene, DNA Star Inc., WI, USA).

### Phylogenetic analyses

Firstly, we prepared the alignment of all the obtained algal nrITS sequences, which we analysed in terms of haplotype variability in FaBox v.1.53 (Villesen 2007). Subsequently, we produced an algal nrITS alignment of the unique haplotypes obtained in this study aligned with the dataset from Muggia et al. (2020) and other recent studies (Xu et al. 2020; Medeiros et al. 2021; Moya et al. 2021; Blázquez et al. 2022; Kosecka et al. 2022) in order to phylogenetically place the photobionts from lichen thalli investigated by the two described methods. Both alignments were performed using MAFFT 7.222 (Katoh & Standley 2013) as implemented in Geneious® 8.0.2 (Biomatters Ltd.), using default options and subsequently refined by hand. Introns and ambiguous regions were excluded from both alignments using Gblocks 0.91b (Castresana 2000) at http://phylogeny.lirmm.fr/phylo_cgi/one_task.cgi?task_type=gblocks using all the options available for the least stringent selection.

The phylogenetic placement of the recovered algal strains was inferred using a maximum likelihood (ML) approach implemented in RAxMLHPC2 8.2.4 (Stamatakis 2014) as implemented in the CIPRES Science Gateway (Miller et al. 2011). The alignment was divided into three partitions: nrITS1, nr5.8S, and nrITS2 regions. The GTR + G nucleotide substitution model was identified as the optimal model under the Akaike Information Criterion (AIC) in jModelTest 2.1.8 (Darriba et al. 2012). Consequently, the GTRGAMMA substitution model was employed for all partitions. We searched for the best scoring ML tree and performed a rapid bootstrap analysis with 1000 pseudoreplicates to assess node support in a single run. We performed a Bayesian analysis in BEAST v 1.10.4 (Suchard et al. 2018) to infer the phylogenetic relationships among our recovered algae. In order to select the best-fit partitioning schemes and nucleotide evolution models, we used PartitionFinder v 2.1.1 (Lanfear et al. 2017). The GTR + G model was used for the nrITS1 and nrITS2, and the HKY + G model was used for the nr5.8S region. We used a coalescent constant size tree prior with a strict clock prior. The MCMC chain length was run during 5 × 10^7^ generations and results were logged every 1000 generations. Trace plots and effective sample sizes (ESS) were examined by TRACER v 1.7 (Rambaut et al. 2018). Finally, after discarding the first 20% sampled trees (burning), the results were summarized and annotated in a maximum clade credibility tree (MCC) through TreeAnnotator v 1.8.4 (Drummond and Rambaut 2007). We the visualization of the ML and Bayesian trees we used Figtree v 1.4.4 (Rambaut 2009) and edited in Adobe Illustrator CC2. Accession numbers for the sequences generated and used during this study are available in Suppl. Table 1.

We assigned names to the new *Trebouxia* lineages discovered during our study according to the most recent classification available on the *Trebouxia* Research Portal (https://Trebouxia.net). This website compiles the most recent phylogenetic framework for this genus (e.g. Leavitt et al. 2015; Muggia et al. 2020; Medeiros et al. 2021, 2025; Barreno et al. 2022; De Carolis et al. 2022; Kosecka et al. 2022; Chiva et al. 2025). Thus, each newly discovered phylogenetic lineage is assigned with a capital letter representing the *Trebouxia* superclade (A, C, D, I, and S), followed by a two-digit number reflecting the order of discovery.

### Photobiont strains and nitrogen treatments

We selected four algal strains (see Results section) from the photobiont screening for physiological studies. Twenty milligrams of each of the selected strains were resuspended in 1 ml of 3NBBM medium. Then, 50 µl of this suspension (c. 1 mg of algal biomass) was inoculated onto 2 × 2 cm sterile nylon membranes. One hundred membranes were inoculated in total per strain. The algal cultures were maintained in agar 3NBBM medium for four weeks under a light intensity of 30 μmol m^−2^ s^−1^ (PPFD) with a 12 h photoperiod at 15 °C. After this period, F_v_/F_m_ and wet biomass were measured in 10 samples of each strain. The remaining membranes were transferred to Petri dishes containing semi-solid medium containing BBM–N (without NaNO_3_) and increasing concentrations of KNO_3_ (0.05 M, 0.1 M, 0.5 M, and 1 M) or (NH_4_)_2_SO_4_ (0.025 M, 0.05 M, 0.25 M and 0.5 M). These concentrations, equivalent to 0.7, 1.4, 7 and 14 g N m^−2^, were chosen considering previous works on the N uptake capacity of lichens (Gaio-Oliveira et al. 2004; Munzi et al. 2009, 2010). In addition, a treatment with 1 M KCl was included to test the possible effect of the osmotic stress caused by high salt concentrations on the photobionts. Each Petri plate contained five biological replicates, and all treatments were performed in duplicate. Lichen photobionts were subjected to the treatments for 12 weeks, after which biomass was measured.

### Photosynthesis measurements

Chlorophyll *a* fluorescence analysis was employed to assess the effect of nitrogen treatments on the different lichen photobionts. The maximum quantum efficiency of photosystem II (PSII), expressed as the F_v_/F_m_ ratio, was measured in samples dark-adapted for 15 min. Measurements were performed weekly over a three-month period using a Handy PEA fluorimeter (Hansatech Instruments), which allows non-invasive readings through the Petri dish lid, ensuring that cultures remained in axenic conditions throughout the experiment.

F_v_/F_m_ was calculated as (F_m_ − F_o_)/F_m_, where F₀ is the minimum and F_m_ the maximum fluorescence emission (Maxwell & Johnson 2000; Govindjee 2004). In the Handy PEA, F₀ is not directly recorded but is estimated a posteriori using an internal algorithm that fits a line to the initial fluorescence rise immediately after the onset of illumination and extrapolates to time zero. Fm is recorded as the peak fluorescence reached during a saturating light pulse of approximately 3,000 µmol photons m⁻^2^ s⁻^1^ applied for 1 s, which fully closes PSII reaction centres. This method provides reliable estimates of both F_o_ and F_m_, enabling accurate calculation of F_v_/F_m_.

### Desiccation experiments

At the end of the nitrogen treatments, the algal cultures were picked from the agar plates, weighed and placed in a desiccator with silica gel (approx. 30% relative humidity). Samples were dried overnight and then were placed on 12 × 12 cm plates on water saturated filter paper for rehydration. The plates were sealed with parafilm and the F_v_/F_m_ parameter was measured after 24 h. The samples were then dried again overnight in the desiccators and then placed in plates sealed with parafilm containing a few grams of silica gel and maintained under growth conditions. At 3, 9 and 15 months, the samples were rehydrated for 24 h and the F_v_/F_m_ parameter was measured, after which they were desiccated again. To ensure that all plates receive the same amount of light throughout the experiment, we systematically rotated their positions each week.

### Statistical analysis

To evaluate the effects of nitrate and ammonium exposure on the maximum photochemical efficiency of PSII (F_v_/F_m_), linear mixed-effects models (LMMs) were fitted separately for two experimental conditions. In both cases, species (four photobiont strains: *T. jamesii*, *T. I01*, *T. gigantea*, *T. A74*) and time (weeks) were included as fixed effects, and replicate was included as a random intercept to account for repeated measures on the same sample.

In the first model, F_v_/F_m_ was analysed as a function of species, nitrate concentration (continuous), and time (continuous). In the second model, nitrogen compound (KCl, nitrate, ammonium) was used as a fixed effect instead of concentration. Both models included all two- and three-way interactions among fixed effects.

Models were fitted in R v4.4.2 using the *lme4* package, with the following specifications:

- Nitrate model: F_v_/F_m_ ~ Species × Concentration × Week + (1 | Replicate)
- Compound model: F_v_/F_m_ ~ Species × Compound × Week + (1 | Replicate)

Significance of fixed effects was assessed using Type III Wald χ^2^-tests (*car* package). Post hoc pairwise comparisons were performed using Tukey-adjusted contrasts (*emmeans* package). Model assumptions were verified by visual inspection of residuals to confirm normality and homoscedasticity. Statistical significance was accepted at *p* < 0.05.

A two-way analysis of variance (ANOVA) was conducted to evaluate the effects of prolonged desiccation and nitrogen pretreatment on F_v_/F_m_. Type III sums of squares were used to estimate the contribution of each factor while controlling for the others. Statistical significance was assessed at *p* < 0.05.

Post hoc comparisons were performed using Fisher’s least significant difference (LSD) test to identify significant pairwise differences between means. All statistical analyses were carried out using *Statgraphics* v19 (StatPoint Technologies, Inc, USA, 2023).

## Results

**Fig. 1:**
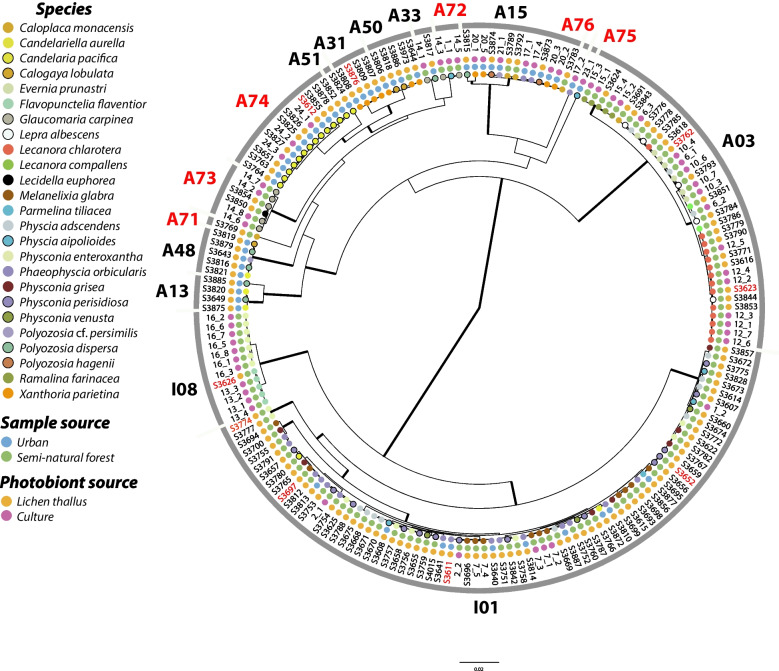
Maximum clade credibility tree obtained in BEAST v 1.10.4, showing the phylogenetic relationships between the haplotypes obtained from the lichen thalli and photobiont cultures. Branches in bold indicate posterior probabilities ≥ 0.95. Circle colours in the first ring represent mycobiont species identities. Second ring represents the source of the sample. Third ring indicates whether the sample was a lichen thallus or a culture. Alphanumeric codes in the subsequent ring represent laboratory codes, with those in red indicating samples from which photobiont cultures were established for that species. Finally, the outermost ring represents the *Trebouxia* lineages according to Muggia et al. (2020). New lineages discovered during this study are provided in red. All information and Gen Bank accession numbers are provided in Suppl. Table 1

We obtained a total of 182 nrITS algal sequences (Fig. 1; Suppl. Table 1), of which 57 were from successful algal cultures from lichen species that were characterised molecularly; 43 were from SN and 14 from U conditions. The remaining 125 sequences (84 SN and 41 U) were obtained from 190 thalli analysed, representing 25 species. All together, we recovered 60 (37 SN, 27 U) different algal haplotypes. We found a high correlation (R^2^ = 0.81) between the number of thalli analysed and the number of haplotypes found in the studied lichen species (Suppl. Table 1). The highest number of haplotypes was found in *Phaeophyscia orbicularis* (7 in 14 studied thalli) and *Candelaria pacifica* (7 in 12 studied thalli). Some species have a lower haplotype number, like *Lecanora chlarotera* and *Lepra albescens*, with two haplotypes each, from six and five thalli respectively. In all but four cases, the strains isolated from a single thallus exhibited identical algal haplotypes. In three cases, *Phaeophyscia orbicularis*, *Physcia biziana* and *Xanthoria parietina*, isolates from a single thallus belonged to two different haplotypes. The case of *Lecanora carpinea* was especially striking since five haplotypes were found in seven isolates from the same thallus. The results of the phylogenetic placement analysis are shown in the outer circle of Fig. 1. The maximum clade credibility tree was based on 8001 trees obtained in the BEAST analyses. All data regarding photobiont diversity are summarized in Fig. 1 and detailed in Suppl. Table 1. The 60 recovered haplotypes corresponded to 14 species-level lineages in the *Trebouxia* A clade and two lineages in clade I according to Muggia et al. (2020) (Fig. 1, Suppl. Table 1). In addition, six of the lineages identified in clade A may represent new species (A71-A76, Fig. 1, Suppl. Table 1). The most common species lineage was I01, to which 74 sequences belonged to. In the second place was the species lineage A03, to which 37 sequences belonged.

We selected four *Trebouxia* species for the nitrogen experiments based on the habitat preferences of their lichen hosts, which were associated with urban environments, semi-natural environments, or both. Among all colonies of the same species, we selected the strain that exhibited the highest growth (indicated in parentheses)*:* 1) An isolate of *Evernia prunastri* (strain 10_7), which was only found in lichen thalli in semi-natural conditions (see below) and identified as *Trebouxia jamesii* (lineage A03 sensu Muggia et al. 2020). 2) An isolate of *Xanthoria parietina* (strain 20_1), found only in lichens growing in urban areas, which was identified as *Trebouxia gigantea* (lineage A15 sensu Muggia et al. 2020), other closely related members of this lineage also occur in semi-natural conditions. 3) An isolate of *Melanelixia glabra* (strain 7_1) found in lichens collected both in semi-natural conditions and from urban areas, which corresponds to the lineage I01 sensu Muggia et al (2020). 4) An isolate from *Candelaria pacifica* (strain 24_1) was found exclusively in thalli of *C. pacifica* growing in the urban area, which seems to belong to a previously unknown lineage in the large clade A sensu Muggia et al. (2020) and which we refer to as *Trebouxia* A74.

### Effect of nitrogen in the culture medium on the photosynthetic performance of isolated photobionts

**Fig. 2:**
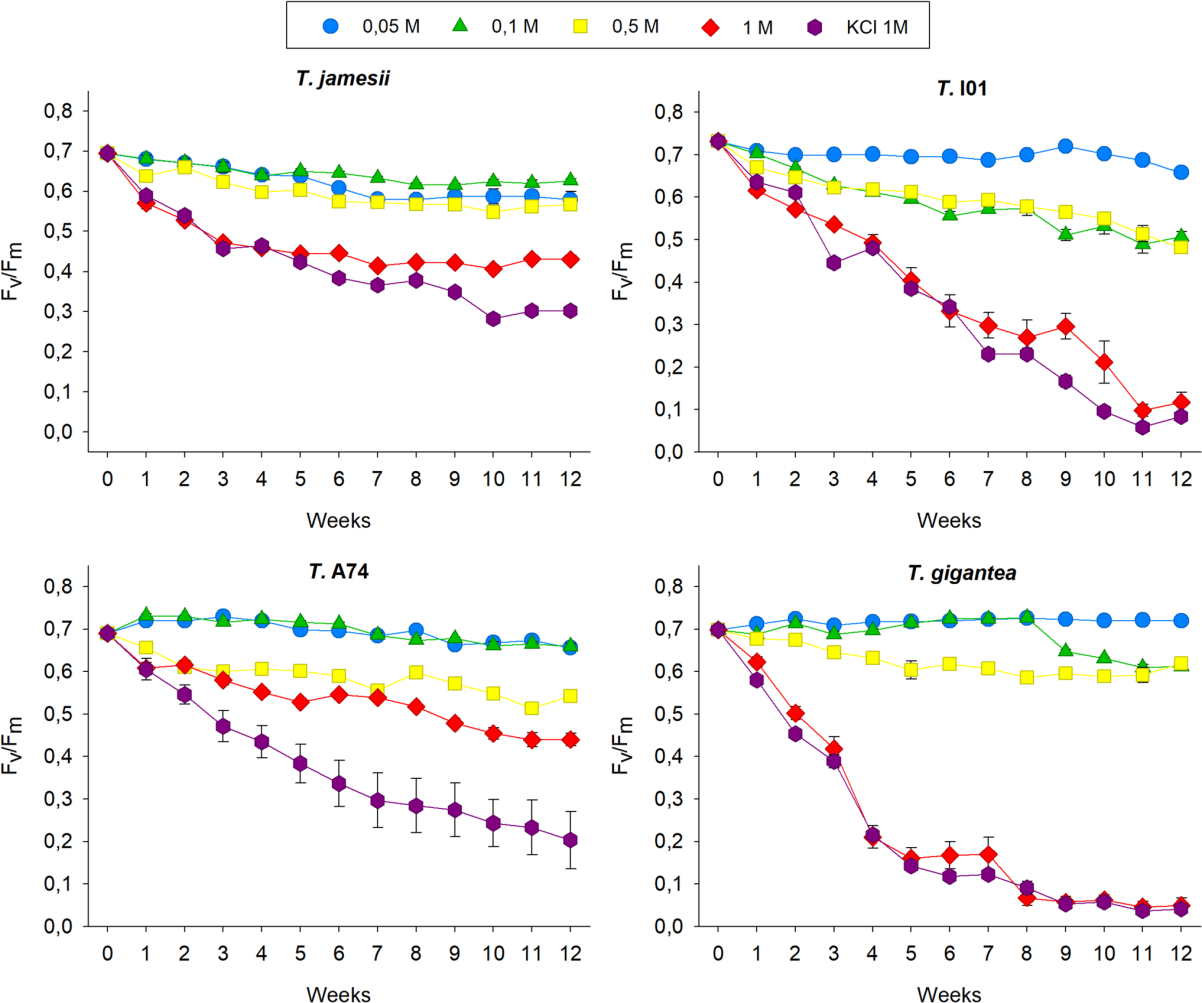
Changes in the maximum photosynthesis yield of the photosystem II (F_v_/F_m_) in the isolated photobionts *Trebouxia jamesii*, *T.* I01, *T.* A74 and *T. gigantea* during 12 weeks of exposure to KNO_3_ 0.05 M (blue circles), 0.1 M (green triangles), 0.5 M (yellow squares), 1 M (red diamonds) and to 1 M KCl (purple hexagons). Symbols represent the average, while bars the standard error of ten biological replicates

The results indicate that while all species show a significant decline in F_v_/F_m_ with increasing nitrate and time, the magnitude of this effect varies. (Fig. 2, Suppl. Table 2 A). In *T. jamesii*, all concentrations of KNO₃ led to a gradual decrease in the maximum photosynthetic yield of photosystem II (F_v_/F_m_) over the first 6–8 weeks of treatment, after which the values stabilized. Concentrations of 0.05, 0.1, and 0.5 M KNO₃ resulted in an approximate 15% reduction in F_v_/F_m_, while a 1 M KNO₃ concentration caused a 39% reduction by the end of the treatment period. In the case of *T.* I01, the effect of the 1 M KNO_3_ treatment was more pronounced, with F_v_/F_m_ dropping from 0.731 to 0.117, representing an 84% decrease, after twelve weeks of exposure. F_v_/F_m_ declined by 35% in the samples treated with 0.1 and 0.5 M, while those exposed to 0.05 M were barely affected. The exposure of *T.* A74 to low concentrations of nitrate (0.05 M and 0.1 M) resulted in a transient increase of F_v_/F_m_ (approximately 5% higher) during the first eight weeks of treatment, after which the values returned to the initial values (0.690). The photosynthesis activity exhibited a general decline across the remaining treatments, with the F_v_/F_m_ values reaching a 37% reduction in the 1 M treatment (0.440), relative to values observed at the commencement of the experiment. In *T. gigantea*, F_v_/F_m_ also increased by approximately 5% in response to the application of 0.05 and 0.1 M KNO_3_. However, while the response to 0.05 M resulted in elevated F_v_/F_m_ values throughout the treatment period, the response to 0.1 M began to decline after 8 weeks, reaching a value of 0.610. The samples subjected to 0.5 M exhibited an 11% reduction in F_v_/F_m_, while photosynthesis activity dropped to 0.049 during 1 M treatment, being 93% lower after 12 weeks. In summary, while all species experienced a decline in photosynthetic performance with increasing nitrate and time, *T.* A74 appeared to be more resilient, whereas *T. gigantea* and, to a lesser extent, *T.* I01 were more negatively impacted (Suppl. Table 2B).

**Fig. 3:**
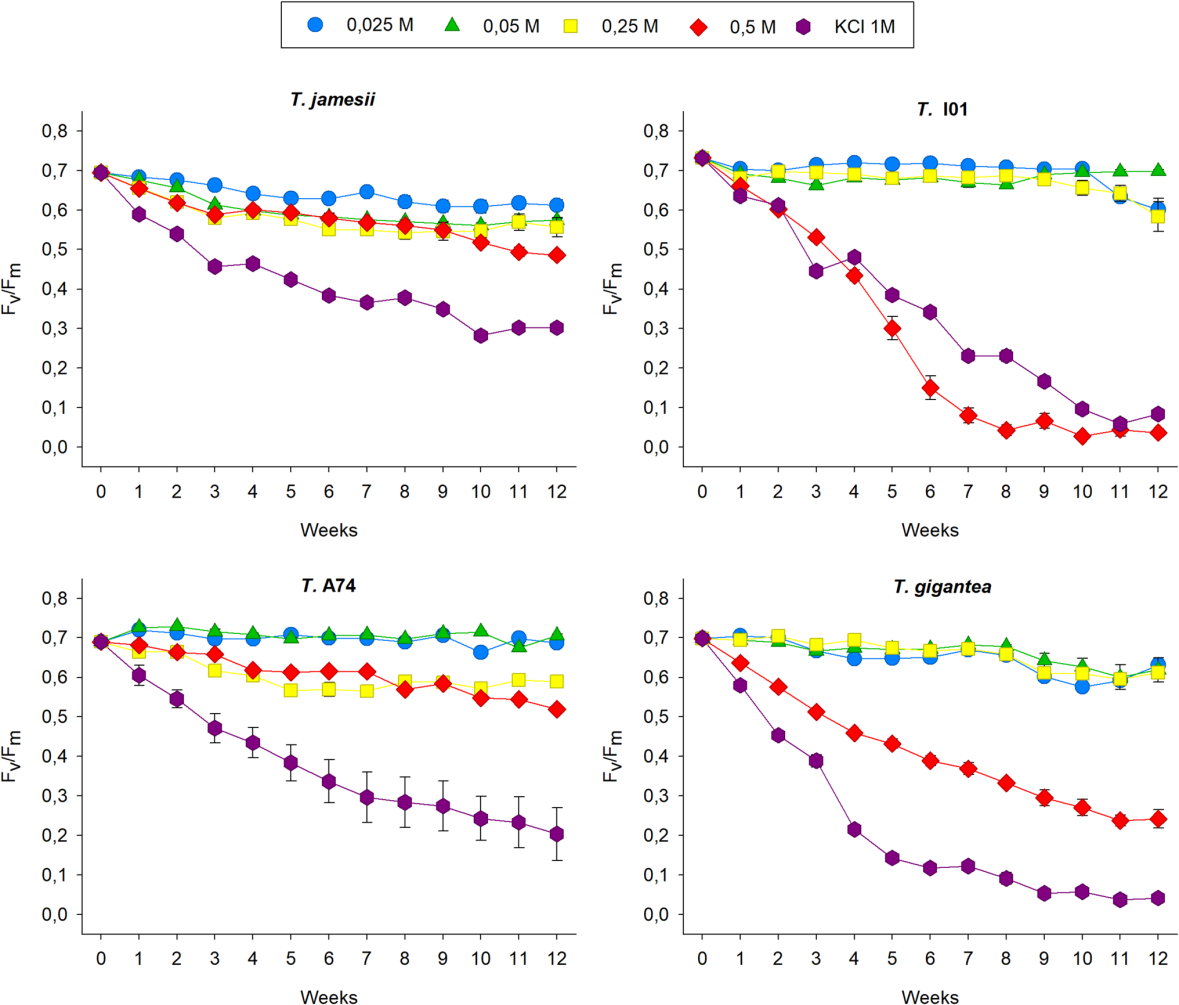
Changes in the maximum photosynthesis yield of the photosystem II (F_v_/F_m_) in the isolated photobionts *Trebouxia jamesii*, *T.* I01, *T.* A74 and *T. gigantea* during 12 weeks of exposure to (NH_4_)_2_SO_4_ 0.025 M (blue circles), 0.05 M (green triangles), 0.25 M (yellow squares), 0.5 M (red diamonds) and to 1 M KCl (purple hexagons). Symbols represent the average, while bars the standard error of ten biological replicates

Ammonium treatment affected the photobionts somewhat differently compared to nitrate, as ammonium concentration alone did not significantly impact F_v_/F_m_, but its effect became significant over time and varied among species (Fig. 3, Suppl. Table 3 A). The trend of F_v_/F_m_ in *T. jamesii* showed a continuous decrease with respect to the initial values in all treatments with (NH_4_)_2_SO_4_, ranging from a 12% lower value at 0.025 M to a 30% lower value at 0.5 M. *T.* I01 maintained high values of photosynthesis activity in the presence of 0.025 M, 0.05 M and 0.25 M of ammonium over a ten-week period. Subsequently, F_v_/F_m_ in 0.025 M and 0.25 M declined to 0.590, representing a 20% reduction in comparison to the initial value. Conversely, F_v_/F_m_ markedly declined to a near-zero level following eight weeks of treatment with ammonium 0.5 M. As observed with nitrate, the exposure of *T.* A74 to low concentrations of ammonium (0.025 M and 0.05 M) resulted in an increase in F_v_/F_m_ of approximately 5%. A similar evolution of F_v_/F_m_ (with respect to equivalent KNO_3_ treatments) was observed for 0.25 and 0.5 M, with a decrease of approximately 20% observed after twelve weeks. *T. gigantea* also maintained the initial F_v_/F_m_ values during the first eight weeks of exposure to 0.025, 0.05 and 0.25 M (NH_4_)_2_SO_4_ before a decrease of approximately 12% was observed. In the case of treatment with 0.5 M ammonium, F_v_/F_m_ exhibited a gradual decline until reaching a value of 0.245 at the end of the experiment, which was 65% lower than the initial value. In conclusion, *T.* I01 was the most negatively affected by increasing ammonium over time, followed by *T. gigantea*, while *T.* A74 was the most tolerant (Suppl. Table 3B).

The exposure of algae to 1 M KCl had no different effect on photosynthesis performance compared to higher nitrate and ammonium concentrations, as the compound alone did not significantly influence F_v_/F_m_ (p = 0.802), indicating no overall difference across treatments (Suppl. Table 4 A). The response of the photosynthesis activity to 1 M KCl was almost identical to that observed for 1 M KNO_3_ in all lichen algae, except *T.* A74, in which nitrate had a lesser negative effect than KCl (Fig. 2). Conversely, 1 M KCl only had the same impact on F_v_/F_m_ as 0.5 M (NH_4_)_2_SO_4_ in *T.* I01. In the remaining photobiont strains, the decline in photosynthesis activity with the highest concentration of ammonium was not as pronounced as with KCl (Fig. 3). *Trebouxia jamesii* was the least affected by exposure to 1 M KCl, maintaining the highest F_v_/F_m_, which was significantly higher than that of *T.* I01, *T.* gigantea, and *T.* A74. In contrast, *T. gigantea* showed the lowest F_v_/F_m_ (Suppl. Table 4B).

### Relative growth

After a four-week growth period in 3NBBM medium (containing 0.0088 M NaNO_3_) supplemented with 1% casein and 2% glucose, the average biomass of each culture was as follows: 20.66 mg for *T. jamesii*, 17.07 mg for *T.* I01, 15.64 mg for *T.* A74, and 15.66 mg for *T. gigantea*. We present the effect of nitrogen treatment on relative growth rate (RGR), which is calculated as the biomass at the end of the N treatment divided by the biomass at the beginning of the N treatment.

**Fig. 4:**
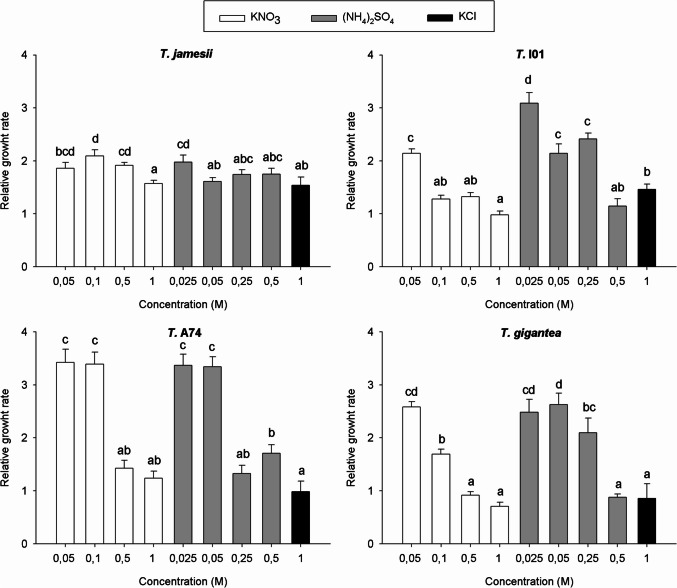
Relative growth rate (final biomass/initial biomass) of the isolated photobionts *Trebouxia jamesii*, *T.* I01, *T.* A74 and *T. gigantea* after 12 weeks of exposure to different concentrations of KNO_3_ (white), (NH_4_)_2_SO_4_ (grey) or KCl (black). Bars represent the average, while standard error of ten biological replicates. Values followed by different letters indicate significant differences (LSD test, 95% confidence values)

As expected, based on their different photosynthesis responses to nitrogen treatments, each photobiont strain exhibited a distinct growth response under identical conditions (Fig. 4). *T. jamesii* exhibited a relatively low growth response to nitrogen treatments, with the biomass increasing between 50 and 100%. The highest RGR (2.09-fold increase relative to the initial values) was observed in the samples exposed to 0.1 M KNO_3_, while the lowest (1.57) was observed in 1 M KNO_3_. *T.* I01 demonstrated a superior response to low and medium ammonium concentrations compared to nitrate. The highest RGR (3.09) was observed in samples exposed to 0.025 M (NH_4_)_2_SO_4_, while the highest RGR with KNO_3_ was 2.15 under 0.05 M. In *T.* A74, low ammonium and nitrate concentrations stimulated growth, resulting in the highest RGR among the four algal strains. The final biomass was approximately three and a half times the initial value in the treatments with 0.05 M and 0.1 M KNO_3_ and 0.025 M and 0.05 M (NH_4_)_2_SO_4_. Similar to *T.* I01, *T. gigantea* showed a superior response to ammonium compared to nitrate, even at the relatively high concentration of 0.25 M (NH_4_)_2_SO_4_.

The exposure to 1 M KCl reduced growth in all four strains (RGR < 1.5), similar to what was observed with the highest concentrations of ammonium and nitrate.

### Long-term desiccation experiment

**Fig. 5:**
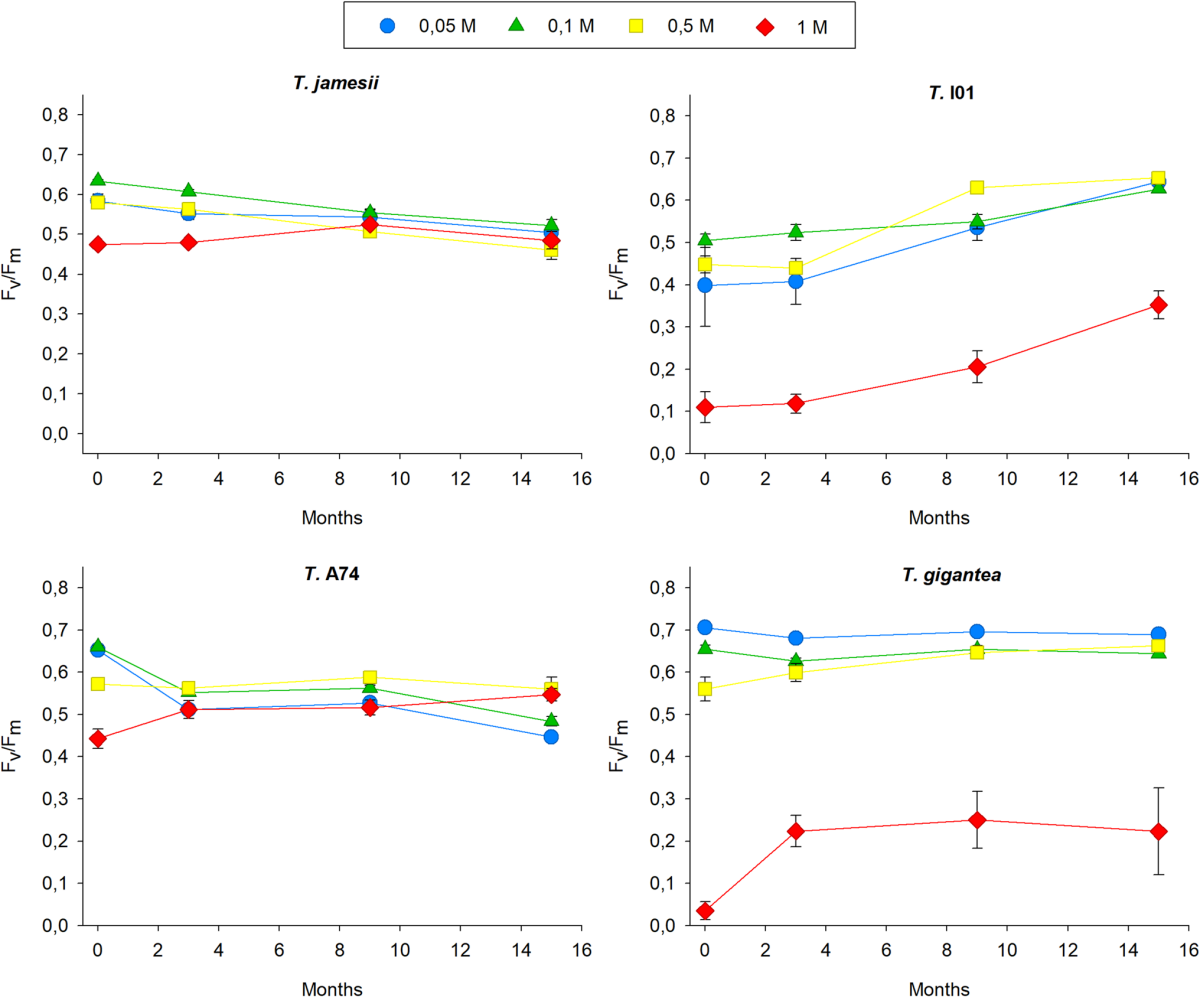
Changes in the maximum photosynthesis yield of the photosystem II (F_v_/F_m_) during long-term desiccation in the isolated photobionts *Trebouxia jamesii*, *T.* I01, *T.* A74 and *T. gigantea* during 12 weeks of exposure to KNO_3_ 0.05 M (blue circles), 0.1 M (green triangles), 0.5 M (yellow squares) and 1 M (red diamonds). Samples were dried immediately after nitrogen treatments and rehydrated the following day, measuring F_v_/F_m_ after 24 h. The following rehydration and desiccation cycles were carried out after 3, 9 and 15 months. Symbols represent the average, while bars the standard error of ten biological replicates

The impact of prolonged desiccation (over a 15-month period, with four 24-h rehydration events at 0, 3, 5, and 15 months) on the nitrate-pretreated samples varied across the different strains and was dose-related (Fig. 5, Suppl. Table 5). In *T. jamesii,* long-term desiccation resulted in a negative impact on the samples pre-treated with 0.05 M, 0.1 M and 0.5 M KNO_3_, as evidenced by a gradual decrease in F_v_/F_m_ values from approximately 0.600 to 0.500. A slight recovery of F_v_/F_m_ was observed in the 1 M pre-treated samples after 15 months of desiccation, with values increasing from 0.430 to 0.485. The photosynthesis activity of *T.* I01 exhibited a notable increase, particularly following the third rehydration (9 months), with the F_v_/F_m_ values reaching approximately 0.650 in 0.05 M, 0.1 M and 0.5 M at the end of the experiments. In 1 M KNO_3_ samples, the F_v_/F_m_ ratio gradually increased, reaching a value of 0.352. The recovery of these samples was unexpected, given that after 12 weeks of exposure to 1 M KNO_3,_ the algal strains had a yellow–brown colour (data not shown) and their F_v_/F_m_ was close to 0.100 (Fig. 2). In the case of *T.* A74, samples exposed to low concentrations of KNO_3_ (0.05 and 0.1 M) exhibited a negative response, while those exposed to higher concentrations (0.5. and 1 M) showed a recovery in photosynthesis activity. Following the desiccation period, samples of *Trebouxia gigantea* exhibited a notable recovery in F_v_/F_m_ values, approaching those observed prior to the N treatments. Samples from the 0.05 M, 0.1 M and 0.5 M treatments demonstrated F_v_/F_m_ values of approximately 0.650. Likewise, samples of *T.* I01 in the 1 M KNO_3_ treatment demonstrated a noteworthy capacity to recover F_v_/F_m_, increasing from 0.043 to 0.223 over a 15-month desiccation period.

**Fig. 6:**
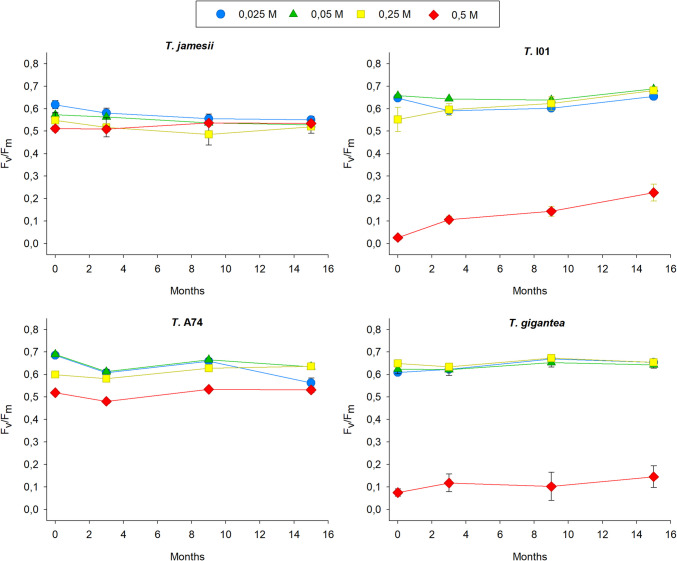
Changes in the maximum photosynthesis yield of the photosystem II (F_v_/F_m_) during long-term desiccation in the isolated photobionts *Trebouxia jamesii*, *T.* I01, *T.* A74 and *T. gigantea* during 12 weeks of exposure to (NH_4_)_2_SO_4_ 0.025 M (blue circles), 0.05 M (green triangles), 0.25 M (yellow squares) and 0.5 M (red diamonds). Samples were dried immediately after nitrogen treatments and rehydrated the following day, measuring F_v_/F_m_ after 24 h. The following rehydration and desiccation cycles were carried out after 3, 9 and 15 months. Symbols represent the average, while bars the standard error of ten biological replicates

The effects of desiccation on the ammonium-pretreated samples of each strain were comparable to those observed for nitrate-pretreated cultures, although less intense (Fig. 6, Suppl. Table 6). In the case of *T. jamesii*, F_v_/F_m_ decreased in the samples pretreated with 0.025 M, whereas in those pretreated with 0.5 M (NH_4_)_2_SO_4_ it increased. *T. I01* also demonstrated a general increase in photosynthesis activity, including the partial recovery of 0.5 M, which had F_v_/F_m_ values close to zero prior to desiccation. *T. A74* showed a slight decrease and increase in F_v_/F_m_ in low and high ammonium concentrations, respectively. *Trebouxia gigantea* maintained the initial values of maximum photosynthesis rate of each (NH_4_)_2_SO_4_ pre-treatment during 15-month desiccation. In conclusion, the F_v_/F_m_ values of the algal strains exposed to the different nitrogen treatments, either nitrate or ammonium, tended to converge to a single value after 15 months of desiccation or after four cycles of desiccation and rehydration in all species. This result indicated that the effects of the nitrogen exposure were likely to have been dissipated during the desiccation experiment, probably due to the washout of salts during the rehydration step.

There are clear differences in the degree of desiccation tolerance of the four algae strains tested when analysing the effect of prolonged desiccation without considering the impact of nitrogen treatments (Table 1). The F_v_/F_m_ values of *T. jamesii* exhibited a continuous decline of 89.0% relative to the initial values observed at the commencement of the desiccation experiment. In contrast, the F_v_/F_m_ values of *T.* I01 and *T. gigantea* were about 30% and 11% higher, respectively. Conversely, the desiccation effect on *T.* A74 was erratic. F_v_/F_m_ decreased by approximately 8% after three months of treatment. This was followed by a recovery to initial values after nine months, and then a further decline of 9% after fifteen months. This decline appeared to be caused by decreased photosynthetic activity observed in samples exposed to low N concentrations.

**Table 1:** Average of the maximum photosynthesis yield of the photosystem II (F_v_/F_m_) during long-term desiccation in the isolated photobionts *Trebouxia jamesii*, *T.* I01*, T.* A74 and *T. gigantea* previously exposed to ammonium and nitrate for 12 weeks (0’). Fluorescence measurements were carried out 24 h upon rehydration, subsequently samples were dried again. Rehydration and desiccation cycles were carried out after 0, 3, 9 and 15 months (Time). Values followed by different letters indicate significant differences (LSD test, 95% confidence values). The *F* values of the ANOVA and the statistical significance (*p* value) are depicted below

Time	*T. jamesii*	*T.* I01	*T.* A74	*T. gigantea*
0'	0.551 c	0.436 a	0.594 b	0.490 ab
0	0.566 c	0.430 a	0.601 b	0.487 a
3	0.546 bc	0.438 a	0.556 a	0.518 abc
9	0.530 ab	0.492 ab	0.587 b	0.548 c
15	0.515 a	0.559 b	0.553 a	0.539 bc
***F*** value	7.03	5.15	9.48	2.54
***p*** value	0.000	0.001	0.000	0.042

## Discussion

The question of which of the lichen symbionts is more sensitive to nitrogen pollution has been a subject of debate in the scientific community over the past few decades (e.g., Hauck 2010; Maslaňáková et al. 2015; Munzi et al. 2017). The findings of our study indicate that, under contrasting levels of nitrogen deposition, not only do mycobiont species differ, but so do the associated photobiont pool. Moreover, the different photobiont strains may be capable of tolerating varying amounts of nitrogen and exhibit different degrees of desiccation tolerance.

### Photobiont diversity

Despite of the significant role of lichens as bioindicators of air pollution in urban environments for over a century (Conti and Cecchetti 2001), and the substantial advances made in the knowledge of photobiont diversity in recent decades (Sanders & Masumoto 2021; Veselá et al. 2024), little is known about the diversity of photobionts in lichens occurring in urban environments, and almost no information is available about how air pollutants affect photobiont diversity. We found species of the genus *Trebouxia* to be the only photobionts present in the lichen species surveyed. Osyczka et al. (2021) reported that *Trebouxia* appears to be particularly well adapted to anthropogenically disturbed environments. In these habitats, some species even replace their typical photobionts from other genera with *Trebouxia* species (Rola et al. 2021). We found more algal haplotypes in the locality with a semi-natural forest (37) than in the urban area (27). However, regarding algal species-level lineages, the urban area presented a more taxa (11) than the semi-natural forest (9). Considering the higher number of mycobiont species surveyed in the latter locality (17) vs the city centre (10), this difference in terms of the number of algal species is surprising. This may be related to the high specialization of species from semi-natural conditions, such as *Lecanora chlarotera* and *Lepra albescens,* which tend to associate with fewer haplotypes. Berlinches de Gea et al. (2024) demonstrated that average mycobiont specialization towards their photobionts tends to increase with better conditions, even within the same mycobiont species. In a study of photobionts associated with lichens growing on heavy metal-polluted areas and areas with no pollution, Bačkor et al. (2010) did not find clear differences in photobiont diversity between polluted and non-polluted areas, even though heavy metal pollution has a larger effect on the photobionts than in the mycobionts (Bačkor et al. 2006; 2007). Thus, considering our results on the molecular diversity of photobionts, it appears improbable that air pollution may cause a steep decrease in photobiont richness, although changes in other facets of diversity should be explored.

While the overall photobiont richness did not differ significantly between the two areas, the composition of the photobiont assemblages was indeed different, with a number of taxa exclusive to either semi-natural or urban conditions. For instance, members of the lineage I01 rarely occur in the city centre, and strains of the lineage A03 were only found in El Pardo. On the contrary, strains of the *Trebouxia* lineages A13, A31, A48, A50, A51, or a previously unknown A lineage were only found in the city centre. Prior research has shown that the photobiont may not be relevant to toxitolerance in *L. conizaeoides* since its photobiont, *T. simplex,* is also present in the pollution-sensitive genus *Pseudevernia* (Hauck et al 2007). Nonetheless, both tolerant and sensitive species are regarded as acidophytic, suggesting that the similar pH of their substrates could explain the sharing of photobionts among these taxa. However, in our case, there is a net difference in nitrogen pollution loads in both areas, and differences in the identity of the algal lineages present in each area are likely to be related to ecophysiological differences between lineages in coping with nitrogen excess (see below). In a recent work on the population structure of the nitrophytic species *Xanthoria parietina* and their associated photobionts using microsatellites in the city of Munich, Wyczanska et al. (2023) observed that the photobionts were highly structured, in contrast to the mycobionts, which showed no clear structure. However, these authors proposed higher dispersal limits between symbionts rather than different pollution levels to explain the differences in population structure. In light of our results, we suggest that future studies should test the role of these two non-exclusive hypotheses.

### Physiology experiments

The experimental investigation of the impact of nitrogen pollution on isolated lichen phycobionts is notably scarce. In one of the few investigations, Marti (1983) exposed cultured photobionts of several epiphytic lichens to aqueous solutions containing different pollutants such as nitrate or sulphite. The impact of these pollutants was assessed by measuring the chlorophyll content and the ^14^C-incorporation rates. In many of the examined species, there was a clear correlation between the sensitivity of the phycobionts and the whole lichen thallus. However, this relationship was not as straightforward in certain species, which showed negative correlations (Marti 1983). Unfortunately, the lichen photobionts were not identified in the aforementioned study, thus precluding a comparison of the results with those obtained in our experiments. Most of the studies comparing the effect of nitrogen pollution on different lichen species have employed the whole thallus as the unit of analysis. Many of these studies have focused on the responses of the species *Evernia prunastri* and *Xanthoria parietina*. *Evernia prunastri* is regarded as one of the most sensitive lichens to nitrogen pollution (De Bakker 1989; Van Herk 1999, 2001), and it is known to always establish symbiosis with the alga *Trebouxia jamesii* (Table 1, Tschaikner et al. 2007). *Xanthoria parietina* is more abundant in environments with high levels of nitrogen deposition (De Bakker 1989; Ruoss 1999; Gaio-Oliveira et al. 2001; Van Herk 2001) and stablishes symbiosis with *T. decolorans*, *T. arboricola*, *T. mariseae* (*T.* A48) and *T. solaris*, all of them close phylogenetic relatives of our *T*. A74 (Nyati et al. 2013, 2014; Muggia et al. 2020). Experiments conducted with transplanted thalli along a NH_3_ field gradient have confirmed that *E. prunastri* is more susceptible (F_v_/F_m_ decrease) than *X. parientina (*Munzi et al. 2014b, 2019). In laboratory conditions, it has been observed that neither species is affected by short-term (30 min to 48 h) exposure to low concentration (0.025–0.050 M) of nitrogen pollutants. However, F_v_/F_m_ decreased rapidly in *X. parietina* and *E. prunastri* when they were exposed to high concentrations (0.5–1 M) (Pirintsos et al. 2009; Munzi et al. 2013). Furthermore, *E. prunastri* shows a detrimental effect of prolonged exposure (three weeks) to low doses of N supply, whereas *X. parietina* was not affected (Munzi et al. 2013). Our results obtained with the isolated photobionts from the thalli of these two species exhibited a high degree of similarity with those observed in the whole thallus. This evidence supports the hypothesis proposed by Munzi et al. (2013) that the tolerance of lichens to N supply is not only species-specific, but also time- and dose-dependent.

Several hypotheses have been put forth to account for the varying degrees of tolerance exhibited by lichens to nitrogen pollution. One of the most widely accepted is that the capacity for cation exchange differs between nitrophytic and acidophilic lichens. Lichens possess many cation exchange sites within the fungal and algal apoplast, primarily comprising of carboxylic and hydroxylic moieties to which diverse N forms can bind (Brown and Brown 1991; Richardson 1985; Casano et al. 2015). Gaio-Oliveira et al. (2001) reported that the cation exchange capacity of *E. prunastri* was approximately five times higher than that of *X. parietina*. Recently, Munzi et al. (2019) demonstrated that the accumulation of ^δ15^N due to NH_3_ supply is threefold higher in *E. prunastri* than in *X. parietina*. Consequently, the high cation exchange capacity of non-nitrophytic lichens, such as *E. prunastri*, might result in nitrogen-sensitive photobionts, like *T. jamesii*, being easily exposed to toxic doses, potentially limiting thallus development in urban environments. In contrast, nitrophytic lichens with low cation exchange capacity, such as *X. parietina*, could shield their photobionts, like *T.* A74, from lethal N concentrations while still allowing for adequate N uptake for optimal growth, although this limited transfer capacity might restrict photobiont development in natural environments with low N inputs.

The N tolerance of some lichens has also been linked to the photobiont’s capacity to provide carbon (C) skeletons and energy for N assimilation. In cells, ammonium is rapidly converted into amino acids to prevent toxic accumulation of free ammonium (Neuhäuser et al. 2007). The nitrate assimilation requires additional reductive power to convert it to ammonium, via nitrite, by the enzyme nitrate reductase. In lichens, photobionts export C to the mycobiont; specifically, *Trebouxia* species export ribitol (Richardson 1985). At non-toxic concentrations, the allocation of N to photobionts increases the photosynthetic capacity and the provision of energy-storing sugars required for basal metabolic processes such as cell repair, growth, and reproduction (Palmqvist et al. 1998; Gaio-Oliveira et al. 2005b; Palmqvist and Dahlman 2006). Throughout our experiments, the photosynthesis activity of *T.* A74, *T.* I01 and *T. gigantea* – all of which were isolated from urban lichens – remained at high levels and, in fact, increased compared to their initial values when they were exposed to low doses of ammonium. Furthermore, low nitrate concentrations also increased F_v_/F_m_ in *T.* A74 and *T. gigantea*. It is probable that the elevated photosynthesis activity observed in these species/treatments probably led to an accelerated growth rate. In the lichenised form, the higher N assimilation and photosynthesis rates of the"urban"photobionts would permit the provision of C skeletons that the mycobiont requires to detoxify the excess N in cities. As the N concentration increases, more carbon is used to assimilate nitrogen and prevent cellular damage in the photobiont. Consequently, fewer carbon skeletons are exported to the mycobiont. As a result of this restriction, the mycobiont becomes more susceptible to high N availability than the photobiont. This hypothesis is supported by several studies, including those conducted by Dahlmann et al. (2002, 2003), Gaio-Oliveira et al. (2004, 2005a), and Munzi et al. (2017). When the limits of photobiont N assimilation are exceeded, the photosynthesis activity can be impaired, thereby reducing the ability of the lichen partners to capture energy and C. This can result in the toxic accumulation of N-salts. The limit of ammonium assimilation was 0.25 M in *T.* I01 and *T. gigantea*, and 0.05 M in *T.* A74, while for nitrate, it was 0.05 M in *T.* I01 and 0.1 M in *T.* A74 and *T. gigantea*. The photosynthesis activity and growth rate of *T. jamesii* declined in response to any nitrogen concentration. This species'low N assimilation capacity may be a contributing factor in why lichens that grow in urban environments do not select this photobiont as a partner.

Another hypothesis put forth by Frahm (2013) is that the “nitrophily” of some lichen species “*is presumably not based upon the facility to higher nitrogen uptake but osmotic tolerance against the salt effects of nitrogen compounds*”. In xeric habitats, such as urban environments, the accumulation of N on the surface of the thallus, and the subsequent dissolution by rain or dew, may result in osmotic restrictions on water uptake. In our experiments with isolated photobionts, we observed a clear similarity between the effect of 1 M KCl and the highest concentration of N compounds, especially with KNO_3_ in *T*. I01 and *T. gigantea*. The evolution of F_v_/F_m_ for 1 M KNO_3_ during the three-week treatment period exhibited a near-identical trajectory to that observed for 1 M KCl. This indicates that the deleterious effect of 1 M KNO_3_ on photosynthesis could be caused by osmotic stress rather than by the toxic effect of N accumulation, supporting Frahm’s hypothesis. However, the anticipated correlation between nitrophily and osmotic stress tolerance was not observed, as the species most severely damaged by 1 M KCl were *T.* I01 and *T. gigantea*, two nitrophytic photobionts, while *T. jamesii*, the most oligotrophic species, was barely affected. As Frahm (2013) outlined, drought-tolerant lichens exhibit a high cellular osmotic pressure, enabling them to absorb water vapour at lower relative humidity levels. This adaptation is believed to be the primary mechanism, with N tolerance being a secondary adaptation. All four *Trebouxia* spp. employed in our study demonstrated strong desiccation-tolerance, as evidenced by their ability to maintain high photosynthetic activity (F_v_/F_m_ > 0.400) after 15 months in a harsh environment (RH < 30%). However, some differences were observed among species. While *T. jamesii* and *T.* A74 exhibited a general decline in F_v_/F_m_ during the prolonged desiccation period, the values for *T.* I01 and *T. gigantea* were higher than at the conclusion of the N treatment. Consequently, the two algae with the lowest osmotic tolerance exhibited the most robust response to desiccation, while the two with the highest tolerance demonstrated the least resilience. It can be concluded, therefore, that osmotic tolerance is not related to desiccation tolerance. However, further experiments are required to determine the capacity of each photobiont to rehydrate using water vapour at different relative humidity levels.

Other authors have proposed that xeric lichens are more prevalent in urban biotas due to their robust cellular protection and repair mechanisms, which enable them to thrive in harsh environments and withstand the toxic effects of N accumulation. Munzi et al. (2013) proposed that *X. parietina* can develop protective mechanisms, initiating rapid repair when growing in the presence of high N availability. In this context, it has been observed that xerophytic species possess more efficient antioxidant and photoprotective mechanisms than hygrophilous ones (Silberstein et al. 1996; Kranner 2002; Weissman et al. 2005), as well as a remarkable regenerative capacity (Honegger 1996). In towns with a dry mesoclimate, the prolonged desiccation results in a depletion of the constitutive levels of scavenging reactive oxygen species and nitric oxide species, accompanied by a reduction in metabolic activity duration. Consequently, hygrophilous species are at a disadvantage compared to xerophytic species, as the former are unable to resist the additional photooxidative stress caused by N pollution and require longer periods of hydration to recover from stress (Tretiach et al. 2012). Among the isolated photobionts, *T.* I01 showed the greatest capacity for recovery. In this species, F_v_/F_m_ increased by approximately 30% during long-term desiccation reaching the pre-N-treatment values. *Trebouxia gigantea* also recovered its highest photosynthetic activity at the end of the desiccation period. Another noteworthy result was that the photosynthesis performance improved in all species treated with 0.5 M ammonium or 1 M KNO_3_, even in those that appeared to have died. It should be noted that algae were only fully hydrated for four days during the 15-months desiccation period, which indicates that *Trebouxia* ssp. have a remarkable capacity to recover after N or osmotic stress. Interestingly, Langmann et al. (2014) also observed that lichens with different N sensitivities recovered quickly during incubation N-free periods. In the experiment above, lichens were subjected to diesel exhaust for five days a week, resulting in a decline of photosynthesis activity (F_v_/F_m_), which was more pronounced in oligotrophic species. However, after nitrogen-free weekends, all species demonstrated a partial recovery of F_v_/F_m_, even in non-nitrophytic lichens. In their study, Langmann et al. (2014) did not explain this phenomenon. However, our interpretation is that it may be more closely related to the capacity of *Trebouxia* algae to recover from osmotic stress than to N toxicity. The significant reduction in photosynthetic performance observed in photobionts exposed to high concentrations of nitrogenous salts is likely due to partial cellular dehydration due to osmotic stress. In both Langmann et al. (2014) and our study, lichens and algae, respectively, were rehydrated with distilled water, a process that would progressively remove excess salts, allowing photobiont rehydration and the rapid recovery of photosynthetic activity. This hypothesis is supported by reports indicating that some lichen species are capable of restoring their photosynthetic activity following acute salt stress when nitrogen is absent or available in low doses (Chowaniec and Rola 2022; Chowaniec et al. 2022, 2023).

## Conclusions

The results of our study demonstrate that *Trebouxia* phycobionts exhibit distinct species-specific physiological traits that are related to their capacity to survive in urban environments.

It can be concluded that the combination and interaction of various ecophysiological traits, including nitrophily, osmotic stress, desiccation tolerance, and recovery capacity, determine the ability of lichen photobionts to survive in harsh and N-polluted cities is. It is evident that the photobiont partner is responsible for providing energy and C skeletons, in the form of carbohydrates, to the mycobiont. Consequently, the sensitivity of the alga may potentially compromise the tolerance of the entire thallus to urban environments.

## Supplementary Information

Below is the link to the electronic supplementary material.

Supplementary file1 (XLSX 26.8 KB)

Supplementary file2 (DOCX 17 KB)

Supplementary file3 (DOCX 17 KB)

Supplementary file4 (DOCX 17 KB)

Supplementary file5 (DOCX 15 KB)

Supplementary file6 (DOCX 15 KB)

## Data Availability

The manuscript has data included as electronic supplementary material.
