# Supplementary material for: Algae and the city: the genetic and ecophysiological diversity of photobionts in two areas of Madrid (Spain) with contrasting levels of nitrogen pollution
Source: Environ Sci Pollut Res Int. 2025 Jul 9;32(30):17978–96. doi: 10.1007/s11356-025-36681-0 (PMC12328547; doi:10.1007/s11356-025-36681-0)
Supplement: Supplementary file 2 — Supplementary file2 (DOCX 17 KB) [file 11356_2025_36681_MOESM2_ESM.docx]

|  | **Chisq** | **Df** | **Pr(>Chisq)** |
| --- | --- | --- | --- |
| (Intercept) | 4380.8 | 1 | <0.001 |
| Species | 20.9 | 3 | <0.001 |
| Concentration | 71.7 | 1 | <0.001 |
| Time | 39.6 | 1 | <0.001 |
| Species:Concentration | 50.9 | 3 | <0.001 |
| Species:Time | 52.7 | 3 | <0.001 |
| Concentration:Time | 2.6 | 1 | 0.107 |
| Species:Concentration:Time | 283.2 | 3 | <0.001 |

**Supplementary Table 2.** Statistical analysis of the effects of nitrate concentration and time on photosynthetic performance in four photobiont strains.

**(A) ANOVA results:** Results of the Type III ANOVA assessing the effects of species, nitrate concentration, and time (weeks) on the maximum photochemical efficiency of PSII (Fv/Fm).

**(B) Post-hoc pairwise comparisons:** Pairwise comparisons between photobiont strains at different nitrate concentrations, adjusted using the Tukey method.

| **contrast** | **estimate** | **SE** | **df** | **t.ratio** | **p.value** |
| --- | --- | --- | --- | --- | --- |
| *T. jamesii - T. I01* | 0.0263 | 0.00396 | 1808 | 6.643 | <.0001 |
| *T. jamesii - T. gigantea* | 0.0194 | 0.00395 | 1808 | 4.914 | <.0001 |
| *T. jamesii - T. A74* | -0.0493 | 0.00388 | 1806 | -12.719 | <.0001 |
| *T. I01 - T. gigantea* | -0.0069 | 0.00400 | 1806 | -1.723 | 0.3117 |
| *T. I01 - T. A74* | -0.0756 | 0.00395 | 1808 | -19.124 | <.0001 |
| *T. gigantea - T. A74* | -0.0687 | 0.00394 | 1808 | -17.432 | <.0001 |
