# Supplementary material for: Algae and the city: the genetic and ecophysiological diversity of photobionts in two areas of Madrid (Spain) with contrasting levels of nitrogen pollution
Source: Environ Sci Pollut Res Int. 2025 Jul 9;32(30):17978–96. doi: 10.1007/s11356-025-36681-0 (PMC12328547; doi:10.1007/s11356-025-36681-0)
Supplement: Supplementary file 3 — Supplementary file3 (DOCX 17 KB) [file 11356_2025_36681_MOESM3_ESM.docx]

**Supplementary Table 3.** Statistical analysis of the effects of ammonium concentration and time on photosynthetic performance in four photobiont strains.

**(A) ANOVA results:** Results of the Type III ANOVA assessing the effects of species, ammonium concentration, and time (weeks) on the maximum photochemical efficiency of PSII (Fv/Fm).

| \|  \| **Chisq** \| **Df** \| **Pr(>Chisq)** \| \| --- \| --- \| --- \| --- \| \| (Intercept) \| 3619.9 \| 1 \| <0.001 \| \| Species \| 29.1 \| 3 \| <0.001 \| \| Concentration \| 0.8 \| 1 \| 0.367 \| \| Time \| 27.0 \| 1 \| <0.001 \| \| Species × Concentration \| 6.3 \| 3 \| 0.097 \| \| Species × Time \| 60.8 \| 3 \| <0.001 \| \| Concentration × Time \| 7.2 \| 1 \| 0.007 \| \| Species × Concentration × Time \| 373.3 \| 3 \| <0.001 \| |
| --- | --- | --- | --- | --- | --- | --- | --- | --- | --- | --- | --- | --- | --- | --- | --- | --- | --- | --- | --- | --- | --- | --- | --- | --- | --- | --- | --- | --- | --- | --- | --- | --- | --- | --- | --- | --- |

**(B) Post-hoc pairwise comparisons:** Pairwise comparisons between photobiont strains at different ammonium concentrations, adjusted using the Tukey method.

| **Contrast** | **Estimate** | **SE** | **df** | **t-ratio** | **p-value** |
| --- | --- | --- | --- | --- | --- |
| *T. jamesii* - *T. I01* | 0.01898 | 0.00415 | 1866 | 4.570 | <0.001 |
| *T. jamesii* - *T. gigantea* | 0.00372 | 0.00415 | 1866 | 0.895 | 0.807 |
| *T. jamesii* - *T. A74* | -0.05868 | 0.00415 | 1866 | -14.129 | <0.001 |
| *T. I01* - *T. gigantea* | -0.01527 | 0.00415 | 1866 | -3.675 | 0.001 |
| *T. I01* - *T. A74* | -0.07767 | 0.00415 | 1866 | -18.699 | <0.001 |
| *T. gigantea* - *T. A74* | -0.06240 | 0.00415 | 1866 | -15.024 | <0.001 |
