# Supplementary material for: Algae and the city: the genetic and ecophysiological diversity of photobionts in two areas of Madrid (Spain) with contrasting levels of nitrogen pollution
Source: Environ Sci Pollut Res Int. 2025 Jul 9;32(30):17978–96. doi: 10.1007/s11356-025-36681-0 (PMC12328547; doi:10.1007/s11356-025-36681-0)
Supplement: Supplementary file 4 — Supplementary file4 (DOCX 17 KB) [file 11356_2025_36681_MOESM4_ESM.docx]

**Supplementary Table 4.** Statistical analysis of the effects of species and time on the maximum photochemical efficiency of PSII (Fv/Fm) under different treatments.

**(A) Type III ANOVA results.** Analysis of deviance (Type III Wald chi-square tests) evaluating the effects of species (*T. jamesii*, *T.* I01, *T. gigantea*, *T.* A74), compound (KCl, nitrate, ammonium), and time (weeks) on the maximum photochemical efficiency of PSII (Fv/Fm).

| **Effect** | **Chi-square** | **df** | **p-value** |
| --- | --- | --- | --- |
| (Intercept) | 257.4 | 1 | < 0.001 |
| Species | 33.6 | 3 | < 0.001 |
| Compound | 0.4 | 2 | 0.802 |
| Time | 47.0 | 1 | < 0.001 |
| Species × Compound | 53.7 | 6 | < 0.001 |
| Species × Time | 42.5 | 3 | < 0.001 |
| Compound × Time | 15.2 | 2 | < 0.001 |
| Species × Compound × Time | 67.7 | 6 | < 0.001 |

**(B) Post-hoc pairwise comparisons under KCl treatment.** Pairwise comparisons between species under the control treatment (KCl), using Tukey-adjusted p-values, to determine significant differences in Fv/Fm.

| **Contrast** | **Estimate** | **SE** | **df** | **t-ratio** | **p-value** |
| --- | --- | --- | --- | --- | --- |
| *T. jamesii* - *T. I01* | 0.10918 | 0.0177 | 2171 | 6.171 | <0.001 |
| *T. jamesii* - *T. gigantea* | 0.21133 | 0.0180 | 2165 | 11.718 | <0.001 |
| *T. jamesii* - *T. A74* | 0.14802 | 0.0176 | 2203 | 8.391 | <0.001 |
| *T. I01* - *T. gigantea* | 0.10215 | 0.0177 | 2171 | 5.774 | <0.001 |
| *T. I01* - *T. A74* | 0.03885 | 0.0166 | 2203 | 2.339 | 0.0898 (ns) |
| *T. gigantea* - *T. A74* | -0.06331 | 0.0176 | 2203 | -3.589 | 0.0019 |
