# Supplementary material for: Algae and the city: the genetic and ecophysiological diversity of photobionts in two areas of Madrid (Spain) with contrasting levels of nitrogen pollution
Source: Environ Sci Pollut Res Int. 2025 Jul 9;32(30):17978–96. doi: 10.1007/s11356-025-36681-0 (PMC12328547; doi:10.1007/s11356-025-36681-0)
Supplement: Supplementary file 5 — Supplementary file5 (DOCX 15 KB) [file 11356_2025_36681_MOESM5_ESM.docx]

**Supplementary Table 5**. F values from the two-way ANOVAs performed on F_v_/F_m_ data of samples pretreated with varying concentrations of KNO_3_ over 15 months (Time) of desiccation. Statistical significance is depicted as * (*p* < 0.05), ** (*p* < 0.01) and *** (*p* < 0.001).

|  | *T. jamesii* | *T.* I01 | *T.* A74 | *T. gigantea* |
| --- | --- | --- | --- | --- |
| Time | 15.9*** | 20,42*** | 14,85*** | 4,40*** |
| Concentration | 50.18*** | 94,03*** | 36,69*** | 268,89*** |
| Time x concentration | 6.11*** | 0,78 | 20,39*** | 2,49** |
