# Supplementary material for: Algae and the city: the genetic and ecophysiological diversity of photobionts in two areas of Madrid (Spain) with contrasting levels of nitrogen pollution
Source: Environ Sci Pollut Res Int. 2025 Jul 9;32(30):17978–96. doi: 10.1007/s11356-025-36681-0 (PMC12328547; doi:10.1007/s11356-025-36681-0)
Supplement: Supplementary file 6 — Supplementary file6 (DOCX 15 KB) [file 11356_2025_36681_MOESM6_ESM.docx]

**Supplementary Table 6**. F values from the two-way ANOVAs performed on F_v_/F_m_ data of samples pretreated with varying concentrations of (NH_4_)_2_SO_4_ over 15 months (Time) of desiccation. Statistical significance is depicted as * (*p* < 0.05), ** (*p* < 0.01) and *** (*p* < 0.001).

|  | *T. jamesii* | *T.* I01 | *T.* A74 | *T. gigantea* |
| --- | --- | --- | --- | --- |
| Time | 1,47 | 3,06* | 25,50** | 1,53 |
| Concentration | 9,80*** | 508,05*** | 221,80*** | 478,94*** |
| Time x concentration | 1,12 | 2,16* | 11,16** | 2,32* |
